# Long COVID Symptomatology After 12 Months and Its Impact on Quality of Life According to Initial Coronavirus Disease 2019 Disease Severity

**DOI:** 10.1093/ofid/ofac397

**Published:** 2022-08-05

**Authors:** Aurélie Fischer, Lu Zhang, Abir Elbéji, Paul Wilmes, Pauline Oustric, Therese Staub, Petr V Nazarov, Markus Ollert, Guy Fagherazzi

**Affiliations:** Deep Digital Phenotyping Research Unit, Department of Population Health, Luxembourg Institute of Health, Strassen, Luxembourg; Bioinformatics Platform, Luxembourg Institute of Health, Strassen, Luxembourg; Deep Digital Phenotyping Research Unit, Department of Population Health, Luxembourg Institute of Health, Strassen, Luxembourg; Luxembourg Center for Systems Biomedicine, University of Luxembourg, Belvaux, Luxembourg; Association “Après J20”, Lucé, France; Service National des Maladies Infectieuses, Centre Hospitalier de Luxembourg, Luxembourg, Luxembourg; Bioinformatics Platform, Luxembourg Institute of Health, Strassen, Luxembourg; Department of Infection and Immunity, Luxembourg Institute of Health, Esch-sur-Alzette, Luxembourg; Department of Dermatology and Allergy Center, Odense Research Center for Anaphylaxis, University of Southern Denmark, Odense, Denmark; Deep Digital Phenotyping Research Unit, Department of Population Health, Luxembourg Institute of Health, Strassen, Luxembourg

**Keywords:** cluster, COVID-19, long COVID, SARS-CoV-2, severity, symptoms

## Abstract

**Background:**

“Long COVID” is characterized by a variety of symptoms and an important burden for affected people. Our objective was to describe long COVID symptomatology according to initial coronavirus disease 2019 (COVID-19) severity.

**Methods:**

Predi-COVID cohort study participants, recruited at the time of acute COVID-19 infection, completed a detailed 12-month symptom and quality of life questionnaire. Frequencies and co-occurrences of symptoms were assessed.

**Results:**

Among the 289 participants who fully completed the 12-month questionnaire, 59.5% reported at least 1 symptom, with a median of 6 symptoms. Participants with an initial moderate or severe acute illness declared more frequently 1 or more symptoms (82.6% vs 38.6%, *P* < .001) and had on average 6.8 more symptoms (95% confidence interval, 4.18–9.38) than initially asymptomatic participants who developed symptoms after the acute infection. Overall, 12.5% of the participants could not envisage coping with their symptoms in the long term. Frequently reported symptoms, such as neurological and cardiovascular symptoms, but also less frequent ones such as gastrointestinal symptoms, tended to cluster.

**Conclusions:**

Frequencies and burden of symptoms present 12 months after acute COVID-19 infection increased with the severity of the acute illness. Long COVID likely consists of multiple subcategories rather than a single entity. This work will contribute to the better understanding of long COVID and to the definition of precision health strategies.

**Clinical Trials Registration:**

NCT04380987.

Since March 2020, the coronavirus disease 2019 (COVID-19) pandemic has disrupted the entire world population. The severe acute respiratory syndrome coronavirus 2 (SARS-CoV-2) virus infected >315 million people and caused >5.5 million deaths worldwide as of January 2022 [1] and, given the current body of knowledge, there are major unsolved questions around the long-term health consequences of COVID-19, which has been termed as “long COVID” by patients themselves to characterize the multisystemic, fluctuant, and debilitating symptoms [2]. Long COVID is defined by the World Health Organization as a post–COVID-19 condition that “occurs in individuals with a history of probable or confirmed SARS-CoV-2 infection, usually 3 months from the onset of COVID-19 with symptoms that last for at least 2 months and cannot be explained by an alternative diagnosis. Common symptoms include fatigue, shortness of breath, cognitive dysfunction but also others which generally have an impact on everyday functioning. Symptoms may be new onset, following initial recovery from an acute COVID-19 episode, or persist from the initial illness. Symptoms may also fluctuate or relapse over time” [3].

Indeed, a fraction of the people with COVID-19 experience continuing effects of the disease, with complaints such as tachycardia, extreme fatigue, and inability to perform daily physical tasks [4]. The signs and symptoms are diverse and related to multiple organs [5]. Between 53 [6] and 203 [7] symptoms have been described in 10 organ systems such as systemic, thorax, neurological, and digestive but also ears/nose/throat, eyes, vascular, hair and skin, or genitourinary. A recent meta-analysis showed that the most prevalent symptoms vary in prevalence over time but the most prevalent have been shown to be fatigue, memory loss, and dyspnea and that the prevalence of long COVID was higher in people hospitalized during the acute phase [8].

Long COVID can affect adults and children and many of them did not return to the same level of work and quality of life as before COVID-19 infection [7].

Both hospitalized and nonhospitalized persons during acute infection are likely to develop long COVID. However, it remains unclear to what extent the persistence of symptoms or the appearance of new symptoms is related or not related to the severity of acute illness [9, 10].

An increasing number of studies report long-term health consequences in asymptomatic or mildly symptomatic patients or hospitalized patients or in mixed cohorts with COVID-19 [11–16].

For all of these reasons, the identification of predictive markers and risk factors of the long-term sequelae of COVID-19 has been defined as a research priority [17].

Our hypothesis is that long COVID symptomatology may differ according to the initial COVID-19 disease severity and that symptoms can cluster and define subtypes of long COVID. To test it, we aimed to (1) provide an overview of the symptoms reported 12 months after the acute infection among a cohort of COVID-19–positive adults in Luxembourg, composed of study participants with various forms of disease severity during the acute illness; (2) perform a co-occurrence analysis of the symptoms; and (3) evaluate the impact of long COVID on quality of life.

## METHODS

### Population and Study Design

Data were obtained from participants from the Predi-COVID study, a prospective hybrid cohort study of persons with a polymerase chain reaction (PCR)–confirmed diagnosis of COVID-19 in Luxembourg. The Predi-COVID study design and analysis plan has been published previously [18]. In brief, all people with a positive COVID-19 PCR test performed by one of the certified laboratories in Luxembourg were contacted by the Health Inspection Department to explain the measures to be respected and ask them if they agree to be contacted for research purposes. If yes, an experienced research nurse from the Luxembourg Institute of Health contacted the person to explain the study objective and procedures and to collect the participants’ consent.

Baseline characteristics were collected at study inclusion, which was performed in the 5 days after the PCR test result. Participants were then followed digitally 12 months after inclusion with self-reported questionnaires on symptoms and quality of life.

### Patient Consent Statement

The written consent of all participants was obtained before inclusion in the study. Data collection in Predi-COVID follows the best practices guidelines from the German Society of Epidemiology [19]. Predi-COVID is registered at ClinicalTrials.gov (NCT04380987) and was approved by the National Research Ethics Committee of Luxembourg (study number 202003/07) and by the Luxembourg Ministry of Health as the authorizing body in April 2020.

### Study Design

This study is an analysis of participants’ symptoms and health status 12 months after the acute infection. All participants included between 1 May and 8 November 2020 were eligible for the present study (N = 539) and were invited to complete a detailed 12-month questionnaire. Among them, 330 completed the questionnaire (response rate of 61.2%). We further excluded 41 participants with incomplete data and the final study population was composed of 289 participants. Characteristics of the respondents were compared to those of the nonrespondents (Supplementary Table 1).

### Symptoms and Quality of Life

**Figure 1.**
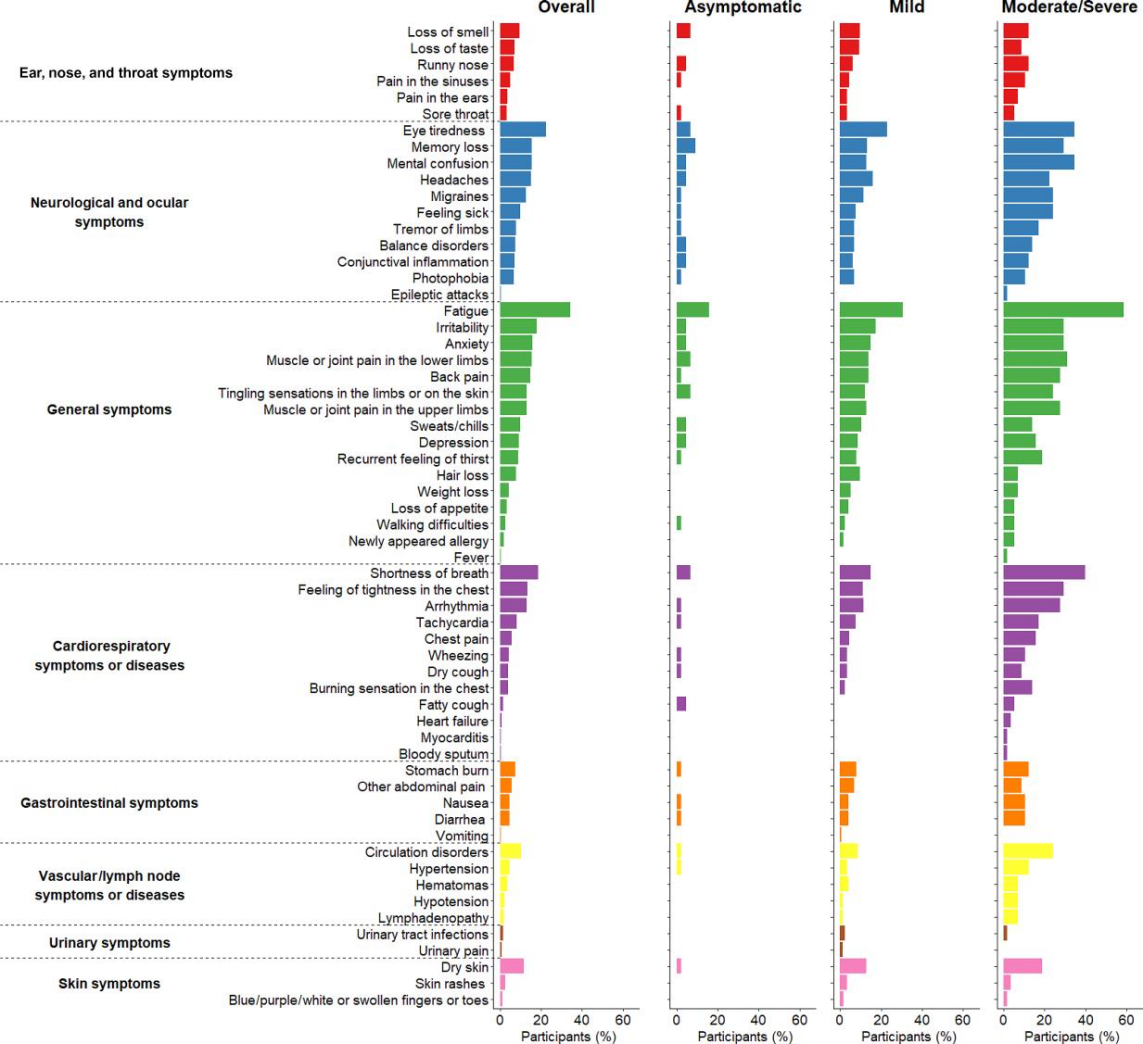
Description of 60 persisting symptoms, 12 months after the acute infection. Symptoms are grouped by symptom category according to disease severity during acute infection. Four symptoms are not presented as no participants reported them: infarction, stroke, hallucinations, and necessity of dialysis.

The detailed 12-month questionnaire was inspired from that co-developed by Tran et al [6] and consisted of a list of the 64 most common symptoms related to long COVID (full list available in Figure 1), classified into 8 main categories: general; ear, nose, and throat; cardiovascular; neurological; gastrointestinal; vascular; urinary; and skin symptoms.

Participants were also requested to answer the question “Have you noticed the following symptoms or illnesses since your COVID-19 diagnosis?” Response modalities were: 1, “Yes, and I still feel it today”; 2, “Yes, I had it but I no longer have it”; and 3, “No, I have never had this symptom.” A variable “total number of symptoms” has been created that corresponds to the sum of all symptoms still present at 12 months.

Sleep quality was assessed using the Pittsburgh Sleep Quality Index (PSQI) questionnaire. A categorical variable was generated using the PSQI score: poor sleep was defined as PSQI total score >5 [20].

The respiratory quality of life was assessed using the VQ11 questionnaire. One global score and 3 subscores (functional, psychological, and relational) were calculated as described elsewhere [21, 22] and categorical variables were generated (Table 1).

**Table 1.** Study Population Characteristics of the Predi-COVID Cohort Study

Characteristic	Overall Population (N = 289)	Disease Severity at Inclusion^a^			*P* Value
		Asymptomatic (n = 44)	Mild (n = 174)	Moderate/Severe (n = 58)	
Age, y, mean ± SD	40.2 ± 12.5	45.4 ± 14.7	39.7 ± 12.1	37.7 ± 11	.006
Female sex	144 (50.2)	13 (29.6)	91 (52.3)	36 (62.1)	.004
BMI, kg/m^2^, mean ± SD	25.6 ± 4.8	25.4 ± 3.5	25.5 ± 4.7	26.1 ± 6.1	.667
Former smoker	55 (19.9)	9 (20.5)	35 (20.1)	11 (19.0)	.925
Current smoker	48 (17.4)	9 (20.5)	30 (17.2)	9 (15.5)	.773
Diabetes	8 (2.9)	1 (2.3)	5 (2.9)	2 (3.5)	1.000
Asthma	8 (2.9)	0 (0.0)	5 (2.9)	3 (5.2)	.382
Cardiovascular disease	9 (3.3)	4 (9.1)	4 (2.3)	1 (1.7)	.065
Hypertension	26 (9.4)	8 (18.2)	13 (7.5)	5 (8.6)	.098
Poor sleep^b^	155 (54.2)	17 (38.6)	93 (54.1)	37 (63.8)	.040
Altered respiratory quality of life^c^ at 1 year	37 (12.9)	0 (0.0)	16 (9.3)	18 (31.0)	<.001
Altered physical autonomy^c^ at 1 year	21 (7.3)	0 (0.0)	7 (4.0)	12 (20.7)	<.001
Altered psychological quality of life^c^ at 1 year	37 (12.9)	0 (0.00)	16 (9.3)	18 (31.0)	<.001
Altered relational quality of life^c^ at 1 year	11 (3.9)	0 (0.00)	2 (1.2)	9 (15.5)	<.001
Could not live in their current health status in the long run	36 (12.5)	4 (9.1)	22 (12.6)	9 (15.5)	.655

Data are presented as No. (%) unless otherwise indicated. *P* values are determined using the analysis of variance significant difference test for continuous variables (age and BMI) and the Fisher exact test for categorical variables.

Abbreviations: BMI, body mass index; SD, standard deviation.

a Information on disease severity at inclusion was missing for 13 participants.

b Sleep quality was assessed using the Pittsburgh Sleep Quality Index (PSQI) questionnaire. A categorical variable was generated using the PSQI score; poor sleep was defined as PSQI total score >5.

c The respiratory quality of life was assessed using the VQ11 questionnaire, initially developed for patients with chronic obstructive pulmonary disease. One global score and 3 subscores (functional, psychological, and relational) were calculated as described elsewhere and categorical variables were generated. An altered respiratory quality of life was defined as VQ11 global score >22, an altered physical autonomy as functional component >8, an altered psychological quality of life as psychological component >10, and an altered relational quality of life as relational component >10.

Finally participants were asked if they could manage their current state of health in the long run, taking into account all the symptoms that they have experienced in the last 30 days in terms of frequency, intensity, and impact on their life and that can be attributed to COVID-19 (yes/no).

### Covariates

Our analysis took into account the following set of covariates: age, sex, body mass index, smoking status (never, former, and current smoker), and comorbidities (diabetes, asthma, cardiovascular disease, and hypertension). The disease severity at inclusion was also used as a potential determinant of developing a more severe form of long COVID.

Disease severity during the acute phase of the disease was defined in 3 categories according to an adapted version of the National Institutes of Health severity classification [23]: asymptomatic, mild illness and moderate/severe illness, as previously described [24]. Hospitalized patients were included in the moderate/severe category.

### Descriptive Statistics

We described the continuous variables, which were normally distributed, as mean ± standard deviation, and categorical variables as numbers (percentage). We used the Student *t* test and 1-way analysis of variance to determine the differences of distribution for continuous variables and Fisher exact test for categorical variables. Kruskal-Wallis test was used to determine the differences for the variable total number of symptoms.

We studied the co-occurrences of long COVID symptoms at 12 months and clustered the symptoms using Ward’s hierarchical clustering method with Euclidean distance [25].

We performed all of the analysis using the R language [26] and generated the figures using the ggplot2 R package [27].

## RESULTS

### Characteristics of Study Participants

Individual characteristics of respondents and nonrespondents to the 12-month questionnaire were similar except for sex, with women being slightly overrepresented in the study population (50.2 vs 40.4%, *P* = .029; Supplementary Table 1). Demographic and clinical characteristics of included participants according to the disease severity during the acute phase are provided in Table 1.

### Description of Persisting Symptoms 12 Months After Acute Infection

Among study respondents with complete data, 172 (59.5%) participants presented at least 1 persisting symptom 12 months after acute infection. Among them, the median total number of persisting symptoms was 6 (IQR, 2–11) and one-third experienced >10 symptoms (Supplementary Table 2).

Four of the 64 symptoms were not reported by any participant (infarction, stroke, hallucinations, and necessity of dialysis). The most frequently reported symptoms were fatigue (34.3%), eye tiredness (22.5%), shortness of breath (18.7%), and irritability (18.0%) (Figure 1).

Participants affected by a moderate or severe acute infection reported more frequently at least 1 symptom 12 months after acute infection in comparison with initially asymptomatic participants who developed symptoms during the follow-up period (82.8% vs 38.6%, *P* < .001), and many symptom frequencies were increased (Supplementary Table 2).

### Co-occurrences

**Figure 2.**
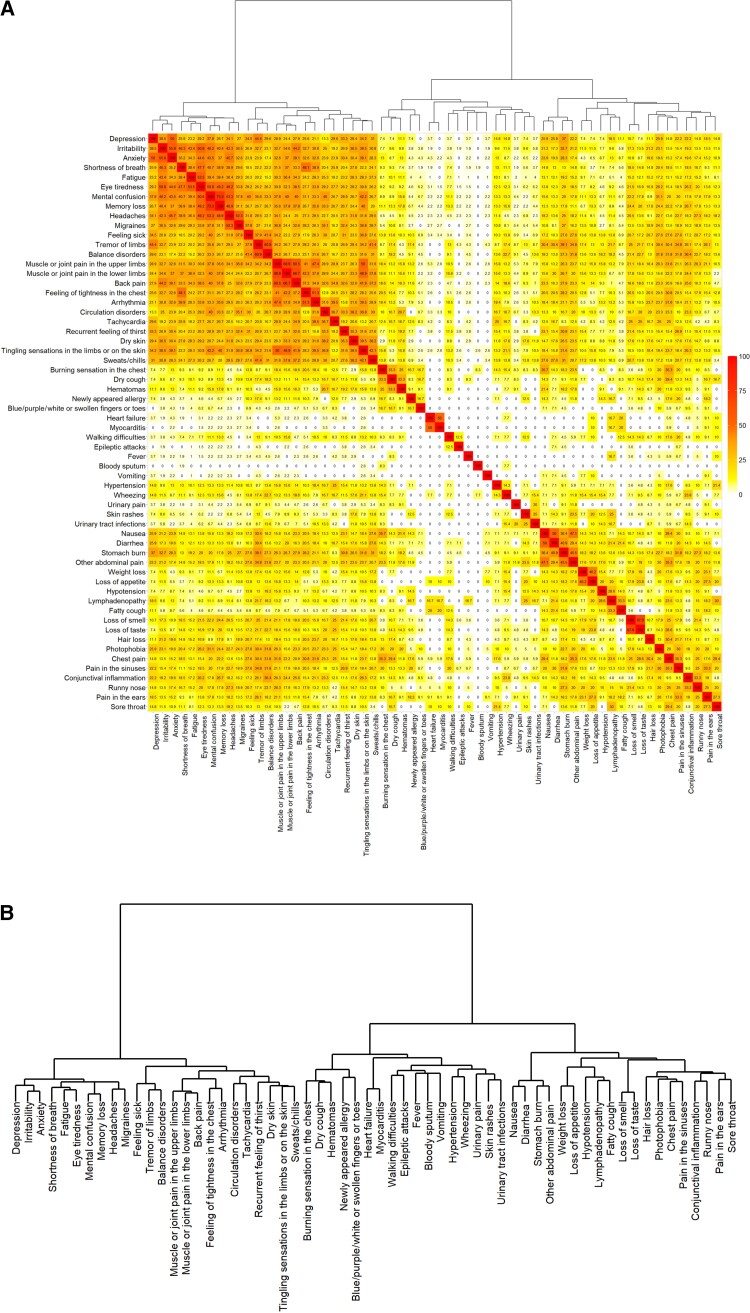
Co-occurrence heatmap of the 60 most frequent symptoms present 12 months after acute infection. *A*, Co-occurrences are presented as percentage per line (eg, line “Depression,” we show the percentage of participants reporting depression and having the second symptom). *B*, Dendrogram indicates the order in which the symptoms were clustered. The smaller height reflects the earlier joining and the higher similarity between the clusters/symptoms.

Figure 2 shows the co-occurrence rates in the 60 persisting symptoms reported by at least 1 participant. The 24 most frequent symptoms, including mainly neurological and cardiovascular symptoms, tended to cluster. A first cluster was mostly constituted by neurological symptoms: When mental confusion was present, memory loss was also reported in 73.3%, headaches in 53.3%, eyes tiredness in 50.8%, irritability in 46.2%, anxiety in 43.5%, shortness of breath in 40.7%, and fatigue in 39.4% of the cases. When balance disorders were present, tremor of the limbs was also reported in 60.9% of the cases. A second cluster was constituted by pain-related and cardiovascular symptoms: Muscle or joint pain in the upper limbs was frequently reported together with muscle or joint pain in the lower limbs, back pain, feeling of tightness in the chest, or arrhythmia (68.9%, 60.5%, 41%, and 47.4%, respectively). Some symptoms were less frequently reported overall, but tended to cluster with each other when present. In particular, gastrointestinal symptoms constituted 1 cluster as 50% of the participants with nausea reported also diarrhea, 36.4% of them had also stomach burns, and 47.1% of them declared other abdominal pain. Finally when loss of taste was present, loss of smell was also reported in 67.9% of the cases.

### Impact on Quality of Life

Poor sleep was reported by 155 (54.2%) participants and 37 (12.9%) participants had an altered respiratory quality of life 12 months after the acute infection. The physical autonomy, psychological, and relational components of the respiratory quality of life score were affected in 7.3%, 12.9%, and 3.9% of the participants, respectively. Participants with a moderate/severe form of the disease during the acute phase reported more frequently poor sleep (63.8% vs 38.6%, *P* = .04) and had a more altered respiratory quality of life (31% vs 0%, *P* < .001) when compared to asymptomatic participants.

Moreover, 36 participants (12.5% of the respondents) answered “No” to the question on whether they could manage their current health status in the long run (Table 1). This represents 20.9% of the 172 symptomatic participants at 12 months. These participants had similar individual characteristics to participants who answered yes to this question (see Supplementary Table 3). All symptom frequencies were increased in participants who declared not being able to cope with their symptoms in the long run compared to the overall study population (Supplementary Table 4).

## DISCUSSION

Our study provides a comprehensive overview of the symptoms reported by people with long COVID 12 months after an acute SARS-CoV-2 infection. We found that almost 60% of the participants experienced at least 1 symptom, with a predominance of fatigue, dyspnea, and anxiety. The presence of symptoms at 12 months was associated with a moderate or severe form of the acute infection. We also observed clusters of symptoms frequently co-occurring and showed that long-term symptoms had a high impact on quality of life.

### Comparison With Literature

In our cohort 59.5% of the participants who fully completed the 12-month questionnaire declared at least 1 symptom 1 year after the acute infection. This proportion was higher for participants with moderate/severe form of acute illness (82.8%) than for participants with mild or asymptomatic initial form of the illness (56.3% and 38.6%, respectively). Other prospective cohort studies report similar frequencies of participants experiencing symptoms 12 months after acute infection [28, 29].

We obtained similar patterns as reported in other studies, with a variety of symptoms and a predominance of fatigue, dyspnea, and anxiety [12, 30–32]. The individual frequencies of these frequent symptoms were similar to their pooled prevalence described in a recent meta-analysis of 12-months follow-up cohort studies; however, those of less frequent symptoms such as loss of taste, loss of smell, or gastrointestinal symptoms were higher in our study [33].

Several mechanisms can explain this wide panel of symptoms: SARS-CoV-2 virus can enter cells of many organs via the ACE2 receptors and thus provoke multiorgan damage. Chronic fatigue can be explained by a dysfunction in inflammatory response pathways, but other factors may be involved too. Neurological and cognitive impairments may be explained by chronic neuronal inflammation and damage since SARS-CoV-2 is able to pass the blood-brain barrier [5]. An acute infection can also induce chronic disturbance in immune subsets or an activation of an autoimmune response, which is in line with the relapses observed in long COVID [34]. In particular autoimmunity can be induced by SARS-CoV-2 virus and is dependent on the initial viral load [32] and the severity of the disease [24].

The total number of symptoms present 12 months after acute infection is difficult to compare with existing studies as the cohort types, the timepoints, and the way to collect symptoms are different [6, 10, 35]. However, it was striking that one-third of the participants of the Predi-COVID cohort who were symptomatic 12 months after the acute infection were experiencing >10 symptoms.

Participants with a moderate or severe form of acute illness had a significantly higher propensity to declare symptoms 12 months later and had a more altered quality of life than those with asymptomatic or mild form of acute illness. Previous studies reported contradictory findings as some studies did not find an association between long COVID and initial disease severity during acute COVID-19 [9, 36], but other studies showed that the severity of the acute COVID-19 illness was associated with an increase in long COVID features [37, 38]. In contrast, we also showed that an initial mild or asymptomatic form of acute disease did not prevent from experiencing long COVID–associated symptoms at 12 months, in particular fatigue, and limited studies are proposing potential mechanisms to explain it [39, 40]. This finding is consistent with a review stating that 30%–60% of patients with an initial asymptomatic or mild form of COVID-19 developed a long COVID [41].

We observed several clusters of symptoms that often co-occurred. Among frequently reported symptoms, we observed a distinct cluster mainly constituted by neurological symptoms and another by pain-related symptoms. Cardiovascular symptoms were present in both clusters but did not constitute a separate cluster. In less frequently reported symptoms, we have seen that gastrointestinal symptoms (nausea, diarrhea, stomach burn, and other abdominal pain) on one hand and loss of taste and loss of smell on the other hand usually co-occurred. This is consistent with findings from other studies confirming that different clusters of long COVID can be described and could benefit from adapted treatments and diagnosis [37, 42].

Symptoms present at 12 months had an important impact on participants’ quality of life, with sleep disturbances, altered respiratory quality of life, and discouragement concerning their state of health. People who experienced a moderate or severe form of initial COVID-19 were the most impacted in terms of sleep and respiratory quality of life. Participants who declared not being able to cope with their symptoms in the long run had similar individual characteristics than those who declared the opposite, and had reported higher symptom frequencies. This further underlines the impact of long-term symptoms on quality of life.

### Strengths and Limitations

Our study has several strengths. First, it is a prospective cohort study including COVID-19 positive persons combining various acute illness stages and followed 12 months after acute infection compared to other studies [12, 43]. All study participants had a PCR-based COVID-19 diagnosis, which avoided the risk of false negatives in the asymptomatic group of participants. Women were slightly overrepresented in the respondent group, which was expected since it has already been shown that women are more frequently affected by long COVID compared with men [44]. We focused our analysis on symptoms present at the time of questionnaire completion to limit the risk of recall bias; as such, it was not possible to clearly describe the dynamics of the various symptoms during the year of follow-up.

This study also has several limitations. The response rate to the long COVID questionnaire was 61.2%, which could lead to an overestimation of symptoms since people without any symptoms might have been less inclined to complete the questionnaire. Participants who responded to the questionnaire had the same individual characteristics as participants who did not respond, which limits but does not completely rule out the risk of selection bias, which could then lead to an overestimation of some symptom frequencies.

We cannot fully state that all symptoms reported in the 12-month questionnaire are associated with COVID-19, in particular for newly appeared symptoms in participants asymptomatic during the acute phase of the infection. However the wording of the question “Have you noticed the following symptoms or illnesses *since your COVID-19 diagnosis*?” limits the risk of reporting symptoms not related to COVID-19. Moreover, the appearance of symptoms in asymptomatic patients or after recovery of the acute illness has been reported by other studies [41, 45]. It can be questionable whether gastrointestinal symptoms could be associated with COVID-19 1 year after infection. A “gastrointestinal post–acute COVID-19 syndrome” has been recently characterized with putative physiological mechanisms including persistent viral antigen in the gut, persistent abnormalities in the mucosa and blood, and/or increase in organ-specific autoantibodies [46].

Symptom intensity was not assessed, except for sleep quality and respiratory quality of life, for which we used validated scales. We did not have the information on potential medication taken by the participants to reduce their symptoms, and this could lead to an underestimation of some symptoms.

Study participants were included before the COVID-19 vaccination campaign started in Luxembourg and thus were not vaccinated at inclusion, with unknown vaccination status after 12 months of follow-up. Therefore, we could not assess the impact of COVID-19 vaccination on the development of long COVID. This would be of high interest to replicate our study as some studies show an association between vaccination and a lower long COVID risk, whereas others have shown no association [47, 48].

## CONCLUSIONS

Our study provides an extensive description of symptoms present 12 months after COVID-19 infection and their impact on the quality of life of COVID-19 patients in a well-characterized prospective cohort. We highlighted a significant burden for people living with long COVID 12 months after the initial infection. An initial moderate or severe illness was associated with an increase in long-term consequences of COVID-19. Finally, our study helps to define long COVID and confirms that it is multisystemic and presents different clusters of symptoms. These results will ultimately help to better identify long COVID in clinical settings and contribute to the definition of precision health strategies.

## Supplementary Material

ofac397_Supplementary_Data

## Supplementary Data

Supplementary materials are available at *Open Forum Infectious Diseases* online. Consisting of data provided by the authors to benefit the reader, the posted materials are not copyedited and are the sole responsibility of the authors, so questions or comments should be addressed to the corresponding author.

## Notes

***Acknowledgments.*** We thank the Predi-COVID participants for their involvement in the study; the members of the Predi-COVID external scientific committee for their expertise; the project team; and the nurses in charge of recruitment, data and sample collection, and management in the field.

***Author contributions.*** A. F., G. F., and L. Z. conceived the idea and designed the study. A. F., G. F., and L. Z. drafted the manuscript. L. Z. and A. F. realized the statistical analysis. G. F. and M. O. obtained funding. A. F. ensured administrative, technical, and material support. All authors contributed to the acquisition, analysis, or interpretation of data. All authors contributed to writing and revised the manuscript for important intellectual content, and approved the final version of the manuscript. A. F. and G. F. have full access to all of the data in the study and take responsibility for the integrity of the data and the accuracy of the data analysis.

***Disclaimer.*** The study funders had no role in the design and conduct of the study; collection, management, analysis, and interpretation of the data; preparation, review, or approval of the manuscript; or decision to submit the manuscript for publication.

***Financial support.*** The Predi-COVID study is supported by the Luxembourg National Research Fund (FNR) (Predi-COVID, grant number 14716273); the André Losch Foundation; and the Luxembourg Institute of Health. The related work of P. W. is supported by the FNR and the European Union Horizon 2020 research and innovation program including the European Research Council and the Luxembourg government.
